# Bright persistent luminescence from pure organic molecules through a moderate intermolecular heavy atom effect†
†Electronic supplementary information (ESI) available: Photophysical properties in solution and crystals, molecular stacking and intermolecular interactions in crystal states, CIE coordinates of crystals and melting point of **CC*n*PhBr**. CCDC 1421285–1421291. For ESI and crystallographic data in CIF or other electronic format see DOI: 10.1039/c5sc03739e



**DOI:** 10.1039/c5sc03739e

**Published:** 2015-11-02

**Authors:** Pengchong Xue, Panpan Wang, Peng Chen, Boqi Yao, Peng Gong, Jiabao Sun, Zhenqi Zhang, Ran Lu

**Affiliations:** a State , Key Laboratory of Supramolecular Structure and Materials , College of Chemistry , Jilin University , 2699 Qianjin Street , Changchun , P. R. China . Email: xuepengchong@jlu.edu.cn ; Email: luran@jlu.edu.cn; b Key Laboratory of Functional Inorganic Material Chemistry (MOE) , School of Chemistry and Materials Science , Heilongjiang University , No. 74, Xuefu Road, Nangang District , Harbin , P. R. China

## Abstract

Generally, strong phosphorescence always implies a short luminescence lifetime. So, pure organic molecules with strong persistent phosphorescence at room temperature are rarely explored. Here, a new strategy is used to explore strong persistent phosphorescence of carbazole derivatives through an intermolecular moderate heavy atom effect. Flexible alkyl chains were inserted between the heavy atom and carbazole moieties so as to avoid a strong internal heavy atom affect. The results indicate that the photoluminescence properties of crystals strongly depend on molecular stacking in crystal states. Moreover, seven molecules among eight carbazole derivatives exhibit persistent room-temperature phosphorescence in their crystals and the *Φ*_P_ values of three compounds exceed 9.5%. Notably, when a 6-(4-bromophenoxy)hexyl group is linked to carbazole, the ambient phosphorescent quantum yield reaches 39.5% and the lifetime is as long as 200 ms. Moreover, it emitted white light with a luminescent efficiency of 72.6% owing to the coexistence of strong fluorescence and phosphorescence.

## Abstract 2

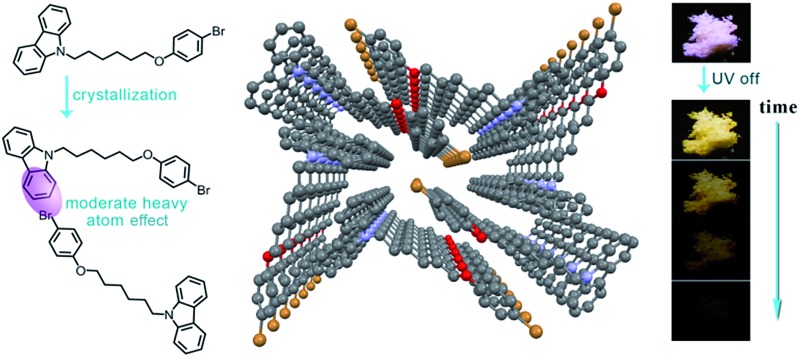
A 6-(4-bromophenoxy)hexyl group linked to carbazole gives crystals that exhibit strong white photoluminescence with an efficiency of 72.6%, a *Φ*_P_ of 39.5%, and a phosphorescence lifetime of 200 ms.

## 

Organic molecules at excited triplet states can be applied to organic synthesis,^1^ bioimaging,^2^ photodynamic therapy,^3^ photo-induced hydrogen production from water,^4^ triplet–triplet annihilation upconversion,^5^ and organic light-emitting diodes.^6^ Pure organic materials hardly demonstrate strong phosphorescence at room temperature because of weak spin–orbital coupling and a long triplet-state lifetime which is easily consumed by rapid nonradiative vibrational relaxation.^7^ Enhanced intersystem crossing (ISC) from singlet excited states to triplet states is required to achieve the visible and efficient phosphorescence of metal-free organic molecules; this process generates molecules at triplet states and shortens phosphorescence lifetime to restrain the effect of molecular vibration on luminescence quenching.^8^ Enhanced ISC can be achieved through strong spin–orbit coupling. Aromatic carbonyls,^9–11^ heavy atom effects,^12–15^ and halogen bonding^16–20^ have been suggested to enhance spin–orbit coupling. For instance, Kim's group found that halogen bonding in co-crystals might activate phosphorescence and result in a phosphorescence quantum yield of 55% and a lifetime of 8.3 ms.^21^ Suppressing the nonradiative decay of triplet states can also efficiently induce pure organic room-temperature phosphorescence (RTP).^22–25^ Tang *et al.* ^26^ suggested that crystallization induces phosphorescence because active intramolecular motions are effectively restricted by the crystal lattice and multiple intermolecular interactions in crystals.

Afterglow or persistent luminescence can be observed when the luminescence lifetime exceeds *ca.* 20 ms because of molecular ultralong-lived excited states. However, persistent RTP is typical in metal inorganic materials, especially in inorganic crystals consisting of rare-earth elements.^27,28^ High phosphorescent efficiencies for organic molecules need strong spin–orbit coupling at room temperature. Thus, bright phosphorescence always implies a short luminescence lifetime (*τ*_P_). Molecules with persistent RTP typically have low phosphorescent quantum yields (*Φ*_P_). For example, we found that 4-(9*H*-carbazol-9-yl)benzaldehyde in a crystal state emitted yellow RTP with a long lifetime of 540 ms, but its *Φ*_P_ is only 9%.^29^ An *et al.* also synthesized a series of phosphorescent carbazole derivatives. The longest *τ*_P_ was up to 1.35 s, but the largest *Φ*_P_ only reached 1.25%.^30^ Li *et al.* reported a carbazole-based benzophenone derivative, whose weak phosphorescence in a co-crystal with chloroform could last more than 1.7 s.^31^ Some deuterated aromatic hydrocarbons embedded in β-estradiol as a host material emit persistent RTP with a lifetime of more than 1 s and a quantum yield of >10%;^32^ however, the development of one-component metal-free organic materials with strong persistent phosphorescence remains a huge challenge. In the present study, we propose a new approach for yielding bright persistent RTP from organic compounds through moderate spin–orbit coupling induced by a moderate heavy atom effect. We deduce that moderate spin–orbit coupling not only promotes efficient ISC from singlet excited states to triplet states (enriching enough molecules at triplet excited states) but also induces a moderate radiative decay rate from triplet states to ground states. Hence, the moderate spin–orbit coupling might balance the values of *τ*_P_ and *Φ*_P_ to afford strong persistent RTP.

Persistent RTP usually could not be detected if a nonmetal heavy atom is covalently linked to a luminophore because of strong spin–orbit coupling.^33,34^ Thus, our designed molecules consist of a luminophore, a flexible chain, and a terminal Br atom (Fig. 1a). Carbazole is selected as the luminophore because of its capacity to stack and form 1D aggregates in crystal form (Fig. 1b) with strong blue fluorescence and detectable weak yellow persistent RTP with a lifetime of 540 ms (Fig. S1†), indicating the existence of weak spin–orbit coupling. Br is selected as the heavy atom because its appropriate heavy atom effect relative to chlorine and iodine may result in moderate spin–orbit coupling.^5^ The introduction of a flexible alkyl chain between Br and a luminophore may avoid an internal heavy atom effect. Thus, heavy atoms will be close to neighboring luminophores in crystal states, a moderate heavy atom effect is anticipated, and persistent RTP is expected. Fig. 1c shows two series of the designed molecular structures (**CC*n*Br** and **CC*n*PhBr**), in which Br and 4-alkoxy-1-bromobenzene serve as the heavy atom groups, respectively. We did not investigate the photophysical properties of **CC3Br** and **CC3PhBr** because they are liquid at room temperature. Moderate heavy atom effects can be found in the crystal states of these derivatives. The *Φ*_P_ values of three compounds (**CC4Br**, **CC4PhBr**, and **CC6PhBr**) exceed 9.5%. Moreover, **CC6PhBr** crystal exhibits strong white photoluminescence (PL) with a total luminescence efficiency of 72.6%, a *Φ*_P_ of 39.5%, and a phosphorescence lifetime of 200 ms. This study is the first to provide an example of an one-component pure organic crystal with such strong room-temperature persistent RTP and white photoluminescence.

**Fig. 1:**
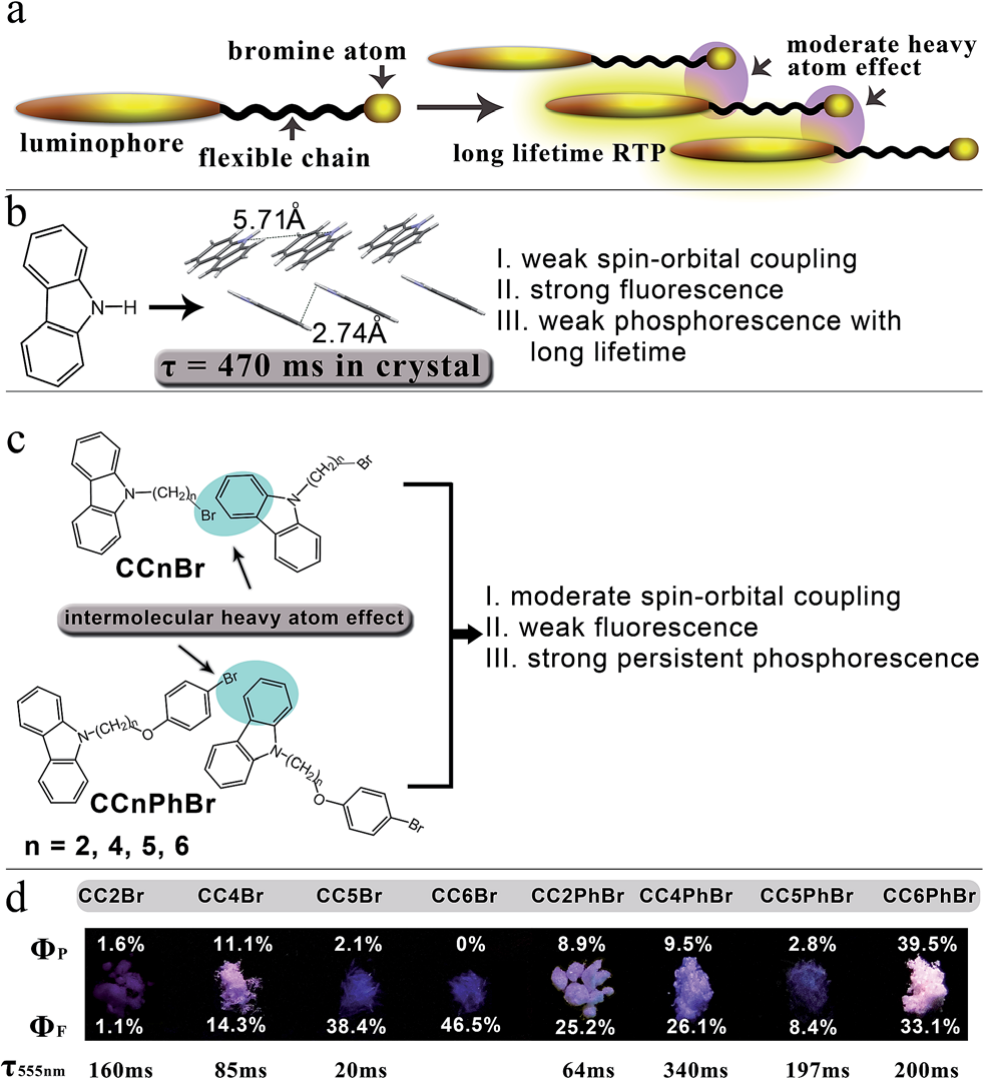
(a) Molecular design principle. The luminophore and heavy atom/group are linked by a flexible chain. (b) Molecular stacking of carbazole in crystals, and the reason for strong fluorescence and detectable persistent phosphorescence with a lifetime of 540 ms is because of weak spin–orbit coupling. (c) Molecular structures of **CC*n*Br** and **CC*n*PhBr** and a description of persistent phosphorescence induced by an intermolecular moderate heavy atom effect. (d) Photos of crystals under 365 nm light with fluorescence and phosphorescence quantum yields and average lifetimes for phosphorescence.

The absorption bands of compounds **CC4Br**–**CC6Br** in toluene are located at *ca.* 332 and 346 nm, and those for **CC2Br** blue-shift to 327 and 341 nm (Table S1 and Fig. S2†) because the electron-withdrawing Br atom is close to carbazole in **CC2Br**. Similarly, **CC2Br** has a blue-shifted emission band with maxima at 341 and 357 nm (Fig. S3†) compared with those for **CC4Br**–**CC6Br** (*ca.* 346 and 362 nm). Moreover, **CC*n*Br** except **CC2Br** shows moderate fluorescence quantum yields (>45%). The short distance between Br and carbazole promotes efficient ISC to quench the fluorescence and yield a low *Φ*_F_ (0.18) of **CC2Br**. These results imply that the internal heavy atom effect disappears when the carbon number of the alkyl chain exceeds 2. **CC4Br** has a strong emission band with a lifetime of 6.75 ns in CH_2_Cl_2_, revealing fluorescence. However, the emission is sharply quenched upon the addition of 1,3-dibromopropane (Fig. S4†), proving that Br can act as an external heavy atom to promote ISC.^35^ Phosphorescence is absent even in neat deoxygenated 1,3-dibromopropane because of the rapid nonradiative vibrational relaxation of long-lifetime triplet states in the solution.

Crystallization may minimize vibrational relaxation and promote Br to be close to neighboring carbazoles, thereby enhancing spin–orbit coupling. Thus, we cultured the crystals of **CC*n*Br** and gained rod-like colorless single crystals (Fig. S5†) that demonstrate distinct photoluminescence behavior. Under irradiation at 365 nm, **CC2Br** crystals afford weak emission, whereas **CC5Br** and **CC6Br** crystals have a strong deep blue emission. Interestingly, white luminescence is observed when **CC4Br** crystals are excited by 365 nm light (Fig. 1d). After the removal of UV light, **CC6Br** turns dark immediately, **CC2Br** and **CC5Br** show weak yellow-green persistent luminescence, and **CC2Br** demonstrates a long persistent lifetime. In particular, strong yellow persistent luminescence can be observed for **CC4Br** crystals. Strong persistent luminescence clearly confirms our molecular design concept. The PL spectra of the crystal states were measured to further investigate the persistent luminescence behavior. **CC5Br** and **CC6Br** have similar emission spectra with a vibrational structure below 500 nm (Fig. 2a). The maximal emission peaks of two compounds are located at *ca.* 370 nm with corresponding short lifetimes of 6.39 and 7.08 ns, revealing their fluorescent nature. Fluorescence quantum yields of **CC5Br** and **CC6Br** crystals in the range of 320–500 nm reached 38.4% and 46.5%, respectively. Additionally, we cannot find phosphorescence from **CC6CBr** crystals, but a weak emission band with a vibrational structure (544 nm, 585 nm and 635 nm) emerges in the PL spectrum of **CC5Br** crystals (Fig. S6†), and its lifetime reaches 20 ms (Fig. 2c). Such an emission is ascribed to phosphorescence,^36^ and the *Φ*_P_ for **CC5Br** crystals is only 2.0%. However, except for the fluorescence emission bands that appear in the range of 350–500 nm with lifetimes less than 3.4 ns, **CC2Br** and **CC4Br** crystals give emission bands with a vibrational structure in the range of 525–700 nm, which originate from phosphorescence because of the long-lived lifetimes. **CC2Br** presents a low luminescence efficiency, an overall *Φ* of 2.7%, and a *Φ*_P_ of 1.6%, although its phosphorescence lifetime is as long as 160 ms. Thus, **CC2Br** is not a good candidate for practical applications. Notably, **CC4Br** crystal possesses a high total *Φ* of 25.3% and a *Φ*_P_ of up to 11% with a long lifetime of 85 ms. The Commission International de I'Eclairage (CIE) coordinates of the **CC2Br**, **CC4Br**, **CC5Br**, and **CC6Br** crystals are (0.31, 0.24), (0.33, 0.30), (0.24, 0.18), and (0.21, 0.16), respectively (Fig. S7, and Table S2†). The CIE points of **CC2Br** and **CC4Br** are located in the white light region. This paper is the first to report metal-free single-component organic crystals with persistent RTP and white photoluminescence.

**Fig. 2:**
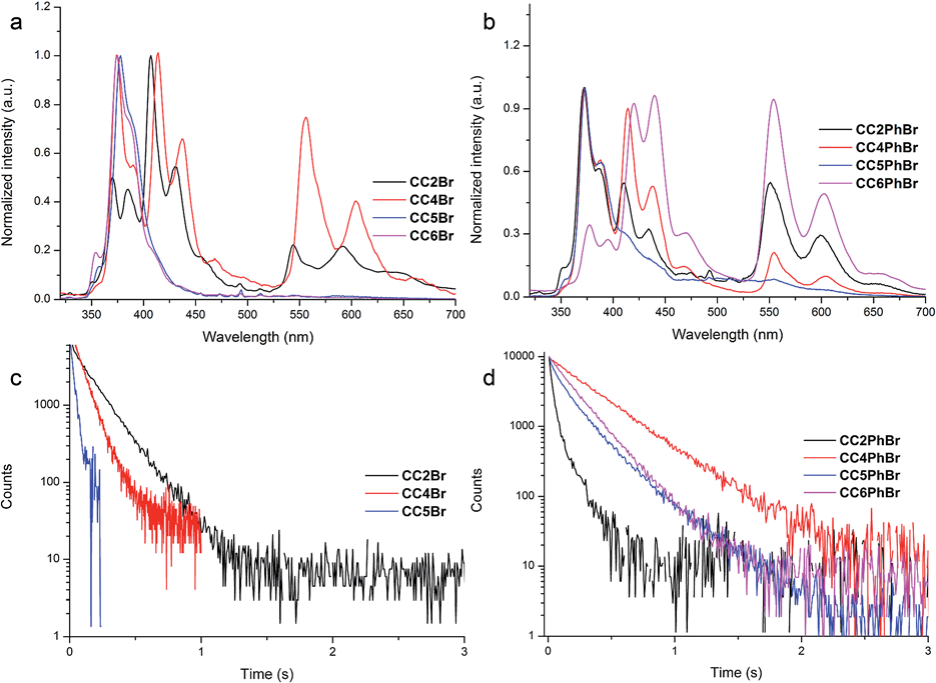
(a) Normalized luminescence spectra of **CC*n*Br** crystals. (b) Normalized luminescence spectra of **CC*n*PhBr** crystals. *λ*_ex_ = 280 nm. (c) Time-resolved measurement of 550 nm emission from **CC*n*Br** crystals. Because of too weak phosphorescence at 550 nm for **CC6Br**, its decay curve was not obtained. (d) Time-resolved measurement of 550 nm emission from **CC*n*PhBr** crystals.

Single-crystal X-ray diffraction was used to reveal the effect of the crystal structures on the luminescence behaviors of **CC*n*Br**. Firstly, the distances between Br and carbazole in crystals are compared because they determine whether RTP happens. An internal heavy atom effect is found in **CC2Br** because the intramolecular Br···N distance is as short as 3.34 Å and the intermolecular distances between Br and the carbons of carbazole exceed 4 Å. Such a short Br···N distance allows a weak electronic interaction between the two atoms and then promotes a moderate ISC rate between singlet and triplet states. Persistent RTP was consequently observed. The intermolecular Br···N distance in the **CC4Br** crystal is 3.74 Å, which is short enough to enhance spin–orbit coupling and allow persistent RTP. By contrast, the Br atom in the **CC5Br** crystal has two orientations and the two Br···N distances are 4.84 and 3.82 Å, which lead to poor spin–orbit coupling. Thus, the **CC5Br** crystal has strong fluorescence and weak phosphorescence. The large Br···N distance (4.47 Å) in the **CC6Br** crystal cannot induce spin–orbit coupling; thus, only fluorescence is detected. Comparative results of the PL spectra and crystal structures show that the emission band around 370 nm should stem from carbazole without the interaction between Br and carbazole, similar to that in **CC6Br**; moreover, the fluorescence around 410 nm and the persistent phosphorescence originate from the carbazoles involved in electronic interactions with the Br atom and carbazole.^16^ These results indicate that the length of the alkyl chain between carbazole and Br may strongly affect the heavy atom effect in the crystals and thus determine whether persistent RTP occurs.^37^ Moreover, it was found that the crystals of 9-ethylcarbazole and 9-butylcarbazole only emitted strong blue fluorescence and no persistent phosphorescence was observed. The PPMA films doped with **CC*n*Br** (5.0% in weight) have similar emission spectra to those in cyclohexane solutions (Fig. S8†). These results indicate that the existence of bromine and the short intermolecular distance between Br and carbazole are critically important for persistent phosphorescence.

The intermolecular stacking and interaction in crystal states were compared to further understand the luminescence properties of different crystals. Four compounds have a similar 1D stacking along carbazole units (Fig. S9†), but the distances between two carbazole moieties are different (Fig. 3, left). The distance of two neighboring carbazoles for **CC2Br** is 3.38 Å, whereas that between two N atoms is 5.42 Å. The two distances in the three other crystals are larger, suggesting a weak π-stacking in the **CC2Br** crystal and that no π-stacking among carbazole moieties in the other three crystals is considered. The change in the UV-vis spectra also supports this conclusion. The absorption band for the **CC2Br** crystal has the largest red shift of 9 nm relative to that in the solution, but less than 5 nm shifts are observed for the three other compounds.^38^ In addition, one molecule of **CC2Br** is only involved in two weak C–H···π interactions (Fig. 3a, right, and Table S3†), which leads to weak suppression of vibrational relaxation. Thus, weak intermolecular interactions and definite π-stacking result in weak PL efficiency for the **CC2Br** crystal.^39^ The **CC4Br** crystal is locked by four intermolecular forces, including two types of C–H···π interactions. Strong intermolecular forces and an absence of π-stacking account for the stronger persistent RTP of the **CC4Br** crystal. Moreover, multiple intermolecular interactions (Fig. 3c and d, right) exist in the **CC5Br** and **CC6Br** crystals, which may cooperate with the lack of π-stacking to allow strong fluorescence.^40^

**Fig. 3:**
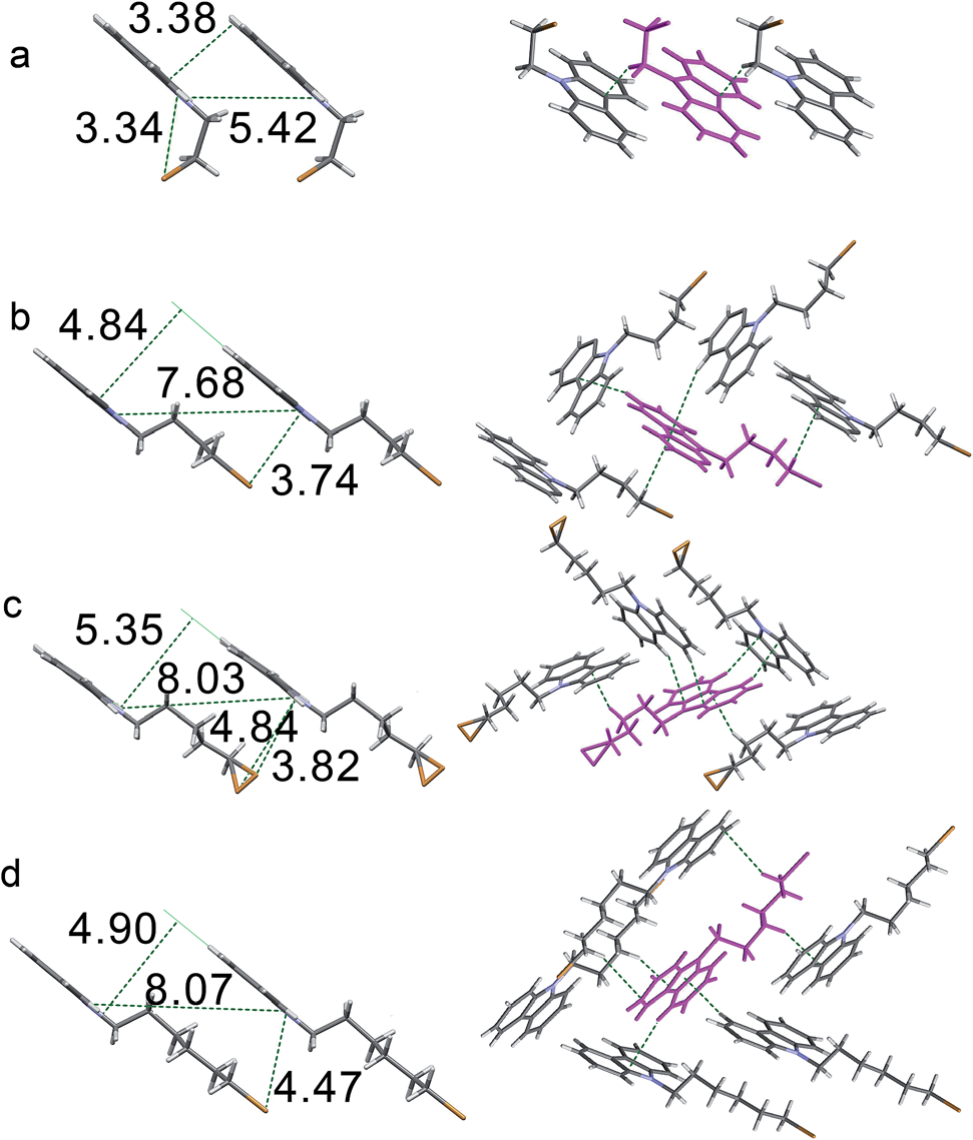
Diagrams of crystal stacking (left) and intermolecular weak interactions (right) in crystals for (a) **CC2Br**, (b) **CC4Br**, (c) **CC5Br** and (d) **CC6Br**. The values are the distances for Br···N, and between two adjacent carbazoles.

The obtained results indicate the importance of intermolecular interactions for enhanced RTP in crystals, meaning that more multiple intermolecular interactions are expected to enhance luminescence efficiency. Therefore, we synthesized a series of compounds, **CC*n*PhBr**, in which 4-alkoxy-1-bromobenzene is used as the heavy atom donor and affords an additional benzene ring to increase intermolecular interactions. The electron-withdrawing characteristic of the oxygen atom leads to blue-shifted absorption and emission bands of **CC2PhBr** relative to other **CC*n*PhBr** compounds in the solutions (Fig. S2 and S3b†). **CC2PhBr**, **CC4PhBr**, **CC5PhBr**, and **CC6PhBr** have similar and moderate fluorescence quantum yields of 43%, 37%, 40%, and 45% respectively in solutions. Similar *Φ*_F_s are ascribed to the long distance between Br and carbazole, and reveal the absence of the heavy atom effect in the solutions. Their fluorescence could also be quenched in the presence of 1,3-dibromopropane. A sample (**CC4PhBr**) is shown in Fig. S4b,† implying that the external heavy atom effect can also efficiently promote the triplet state generation of this series of molecules.

**CC*n*PhBr** compounds also yield different fluorescence properties in crystals (Fig. 1d). The **CC2PhBr** crystal emits white light with a total *Φ* of 33.1% when irradiated at 365 nm. Notably, a very strong white light with a high total *Φ* of 72.6% can be observed for the **CC6PhBr** crystal under UV light. The **CC4PhBr** crystal emits bright blue light with a medium *Φ* of 35.6%. Sheet **CC5PhBr** crystal has a weak emission with a total *Φ* of 11.2%. All four compounds have a yellow persistent RTP with different intensities after the excitation light turns off. The *Φ*_P_ values of **CC2PhBr**, **CC4PhBr**, **CC5PhBr**, and **CC6PhBr** are 8.9%, 9.5%, 2.8%, and 39.5%, respectively, and their corresponding *Φ*_F_ values are 25.2%, 26.1%, 8.4%, and 33.1%. To the best of our knowledge, the **CC6PhBr** crystal possesses the largest persistent phosphorescence quantum yield among organic molecules. Fig. 2b shows the luminescence spectra of the **CC*n*PhBr** crystals. Similar to **CC4Br**, all **CC*n*PhBr** crystals have three groups of emission bands with a vibrational structure, indicating the existence of a moderate heavy atom effect in the four crystals. The fluorescence emission band around 415 nm for **CC4PhBr** is stronger than the phosphorescence, which explains the blue emission color with a CIE coordinate of (0.24, 0.16). The **CC2PhBr** and **CC6PhBr** crystals have two emission bands with almost equal intensities above 400 nm. Their CIE coordinates, (0.30, 0.27) and (0.31, 0.28) respectively, are located at the white light region (Fig. S7 and Table S2†). As shown in Fig. 2d, **CC2PhBr**, **CC4PhBr**, **CC5PhBr** and **CC6PhBr** have lifetimes of 64 ms, 340 ms, 197 ms and 200 ms, respectively. Thus, replacing Br with 4-alkoxy-1-bromobenzene as the heavy atom group supplies an excellent phosphor with strong white light emission and bright persistent RTP.

Single-crystal structures were analyzed to clarify their RTP behaviors. As shown in Fig. 4, the distances between the Br and carbon atoms of carbazole in all crystals are less than 3.90 Å, which ensures that spin–orbit coupling is strong enough to promote efficient ISC. Therefore, the four crystals can emit persistent RTP. π-Stacking is lacking in the four crystals because of long distances between carbazoles (Fig. S10†). Small red-shifts (less than 4 nm) in the UV-vis spectra after crystallization from their solutions may support this result (Table S1 and Fig. S1†). Therefore, the quenching of excited states caused by π-stacking should not be the reason for the distinct solid-state PL. As shown in Fig. 4 and Table S4,† only two short C–H···C distances ascribed to weak C–H···π interactions in the **CC4PhBr** crystal can be observed; thus, this crystal exhibits weak RTP. One **CC6PhBr** molecule can participate in 14 intermolecular interactions with six other **CC6PhBr** molecules (Fig. 4d, right). Multiple strong C–H···π interactions with short distances of less than 2.80 Å are found. In addition, C···C and C–H···H–C interactions work cooperatively to hamper carbazole from undergoing conformational changes and to suppress nonradiative relaxation. Consequently, strong luminescence and bright persistent RTP for **CC6PhBr** crystal are observed.

**Fig. 4:**
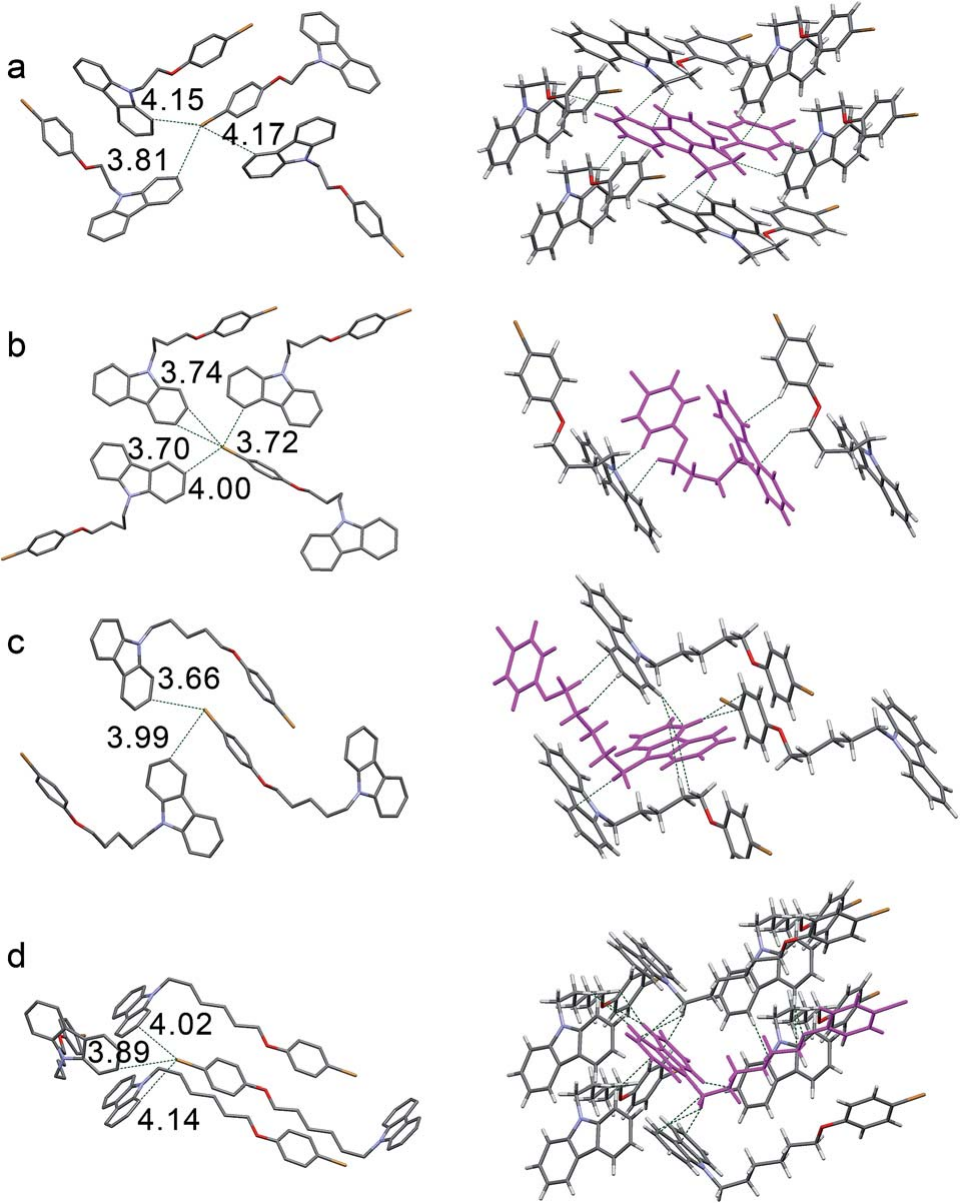
Diagrams of short interactions between the bromine atom and carbon atoms of carbazole (left) and intermolecular weak interactions (right) in crystals of (a) **CC2PhBr**, (b) **CC4PhBr**, (c) **CC5PhBr** and (d) **CC6PhBr**.

**CC2PhBr** is rigidified by multiple interactions with six other molecules (Fig. 4a, right). These interactions should promote high luminescence efficiency similar to that of **CC6PhBr**, but the **CC2PhBr** crystal only possesses weak phosphorescence. Careful observation of this crystal structure shows a large dihedral angle of 5.80° between two phenyl groups of a carbazole unit (Fig. S10†) and small angles of 1.18°, 1.20°, and 2.26° for **CC4PhBr**, **CC5PhBr**, and **CC6PhBr**, respectively. Such a large dihedral angle for **CC2PhBr** generates a non-coplanar structure and may promote low phosphorescence efficiency. Similar to **CC2PhBr**, **CC5PhBr** shows weak emission, although various secondary bonding interactions exist. Molecular conformation is not considered to explain weak emission because the dihedral angle between the two phenyl groups of carbazole is only 1.20° in **CC5PhBr**. The compounds with an alkyl chain with an odd carbon number always have low melting points because of low crystallization tendency or poor compact stacking. For example, **CC3Br** and **CC3PhBr** are liquid at room temperature. **CC5PhBr** hardly crystallizes and has the lowest melting point among the **CC*n*PhBr** compounds (Fig. S11†), indicating weak intermolecular interactions. Moreover, the distances of C–H···C are comparatively longer (more than 2.813 Å), implying weak C–H···π hydrogen bonding and supporting the low melting point. In addition, the alkyl chain does not adopt an all *trans* form, and two small torsion angles (64.07° and 70.33°) exist, which suggests a high molecular energy. As a result, these synergistic effects promote a low quantum yield of **CC5PhBr**.

Due to their strong persistent RTP, **CC4PhBr** and **CC6PhBr** may be used as anti-forgery materials. As shown in Fig. 5, anti-forgery stamps in a common receipt emit red fluorescence under 365 nm light, which immediately disappears when the light is turned off. On the other hand, the patterns of **CC4PhBr** and **CC6PhBr** have strong luminescence with different colors under UV light irradiation. After switching off the excitation source, the two patterns still emit strong yellow phosphorescence, which would gradually disappear.

**Fig. 5:**
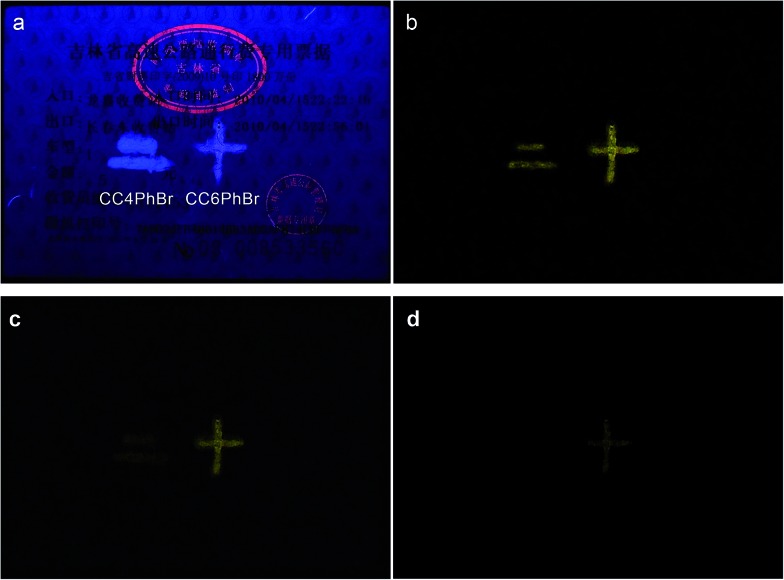
A receipt containing an original anti-forgery stamp with red fluorescence and patterns drawn by the acetone solutions of **CC4PhBr** and **CC6PhBr**. The receipt is under 365 nm light (a), and after the light is turned off for 0.3 s (b), 0.6 s (c) and 0.9 s (d).

In summary, to provide an intermolecular moderate heavy atom effect on phosphorescence, two series of carbazole derivatives with a bromine atom were synthesized, in which Br and carbazole were linked by flexible alkyl chains. The results showed that the distance between the bromine atom and carbazole determined whether room-temperature phosphorescence occurred, and weak intermolecular interactions may strongly influence the luminescence efficiency. Consequently, one metal-free organic crystal with white photoluminescence (*Φ* = 72.6%) and strong room-temperature persistent phosphorescence (*Φ*_P_ = 39.5%, *τ* = 200 ms) was obtained. Such phosphors could act as anti-forgery materials and be applied in document security. We believe that the moderate intermolecular heavy atom effect is an efficient strategy to supply more organic materials with strong room-temperature persistent phosphorescence for real applications. Further work will focus on the introduction of functional units with aggregation-induced emission properties in order to obtain strong persistent phosphorescent materials with different emission wavelengths.
