# Correction: Bright persistent luminescence from pure organic molecules through a moderate intermolecular heavy atom effect

**DOI:** 10.1039/c7sc90048a

**Published:** 2017-07-19

**Authors:** Pengchong Xue, Panpan Wang, Peng Chen, Boqi Yao, Peng Gong, Jiabao Sun, Zhenqi Zhang, Ran Lu

**Affiliations:** a State Key Laboratory of Supramolecular Structure and Materials , College of Chemistry , Jilin University , 2699 Qianjin Street , Changchun , P. R. China . Email: xuepengchong@jlu.edu.cn ; Email: luran@jlu.edu.cn; b Key Laboratory of Functional Inorganic Material Chemistry (MOE) , School of Chemistry and Materials Science , Heilongjiang University , No. 74, Xuefu Road, Nangang District , Harbin , P. R. China

## Abstract

Correction for ‘Bright persistent luminescence from pure organic molecules through a moderate intermolecular heavy atom effect’ by Pengchong Xue *et al.*, *Chem. Sci.*, 2016, DOI: 10.1039/c5sc03739e.



## 

In the original paper, it was proposed that a series of carbazole derivatives with a bromine atom may emit strong persistent room-temperature phosphorescence (RTP) in the crystal state. However, the authors later found that further purification of these carbazole derivatives led to the disappearance of the yellow persistent RTP. Therefore, a small amount of impurities appears to be responsible for the long-wavelength RTP.

The Royal Society of Chemistry apologises for these errors and any consequent inconvenience to authors and readers.

