# Supplementary material for: Bright persistent luminescence from pure organic molecules through a moderate intermolecular heavy atom effect
Source: Chem Sci. 2015 Nov 2;8(9):6060–5. doi: 10.1039/c5sc03739e (PMC5625585; doi:10.1039/c5sc03739e)
Supplement: Supplementary file 3 [file SC-009-C5SC03739E-s003.pdf]

Electronic Supplementary Material (ESI) for Chemical Science.
This journal is © The Royal Society of Chemistry 2015

Bright persistent luminescence from pure organic molecules through moderate intermolecular heavy atom effect

Pengchong Xue,[1*] Panpan Wang,[1] Peng Chen,[2] Peng Gong,[1] Zhenqi Zhang,[1] Jiabao Sun,[1] Ran Lu[1*]

[1] State Key Laboratory of Supramolecular Structure and Materials, College of Chemistry, Jilin University, No. 2699, Qianjin Street, Changchun, P. R. China. Email: xuepengchong@jlu.edu.cn; luran@jlu.edu.cn
[2] Key Laboratory of Functional Inorganic Material Chemistry (MOE), School of Chemistry and Materials Science, Heilongjiang University, No. 74, Xuefu Road, Nangang District, Harbin, P. R. China

Experimental section 3
Fig. S1. 6
Table S1. 7
Fig. S2. 8
Fig. S3. 9
Fig. S4. 10
Fig. S5. 11
Fig. S6. 12
Table S2. 13
Fig. S7. 14
Table S3. 15
Fig. S8. 16
Fig. S9. 19

Experimental section

All reagents were used as purchased without additional purifications. Detail synthesis description sees the Supplementary Information. The UV-vis spectra were determined on a Mapada UV-1800pc spectrophotometer. C, H, and N elemental analyses were performed on a Perkin-Elmer 240C elemental analyzer. Fluorescence measurements were taken on a Shimadzu RF-5301 Luminescence Spectrometer. The fluorescence quantum yields of **CCnBr** and **CCnPhBr** in different solvents were measured by comparing to standards (anthracene in ethanol, $\Phi_F = 0.27$, $\lambda_{ex} = 323$ nm). Photoluminescence spectra and the absolute photoluminescence quantum yields were measured on an Edinburgh FLS920 steady state spectrometer using an integrating sphere. Luminescent decay experiments were measured on an Edinburgh FLS920 spectrometer. EPLED-360 picosecond flash lamp with 898 ps pulse duration and μF920 microsecond flash lamp (pulse width < 2 μs) were used to measure time-resolved fluorescent and phosphorescent spectra, respectively. ^{1}H NMR spectra were recorded on Mercury plus 400 MHz.

Single crystals of **CCnBr** and **CCnPhBr** were obtained by slowing solvent evaporation and selected for X-ray diffraction analysis on in a Rigaku RAXIS-RAPID diffractometer using graphite-monochromated Mo-K_α radiation ($\lambda = 0.71073$ Å). The crystals were kept at room temperature during data collection. The structures were solved by the direct methods and refined on F2 by full-matrix least-square using the SHELXTL-97 program. The C, N, O and H atoms were easily placed from the subsequent Fourier-difference maps and refined anisotropically. CCDC 1421286, 1421288, 1421290, 1421285, 1421287, 1421289, and 1421291 contains the supplementary crystallographic data for **CC4Br, CC2Br**, **CC5Br**, **CC6Br**, **CC2PhBr**, **CC4PhBr**, **CC5PhBr**, and **CC6PhBr**, respectively. The single-crystal structure of **CC2Br** has been reported.[1]

Synthesis of compounds

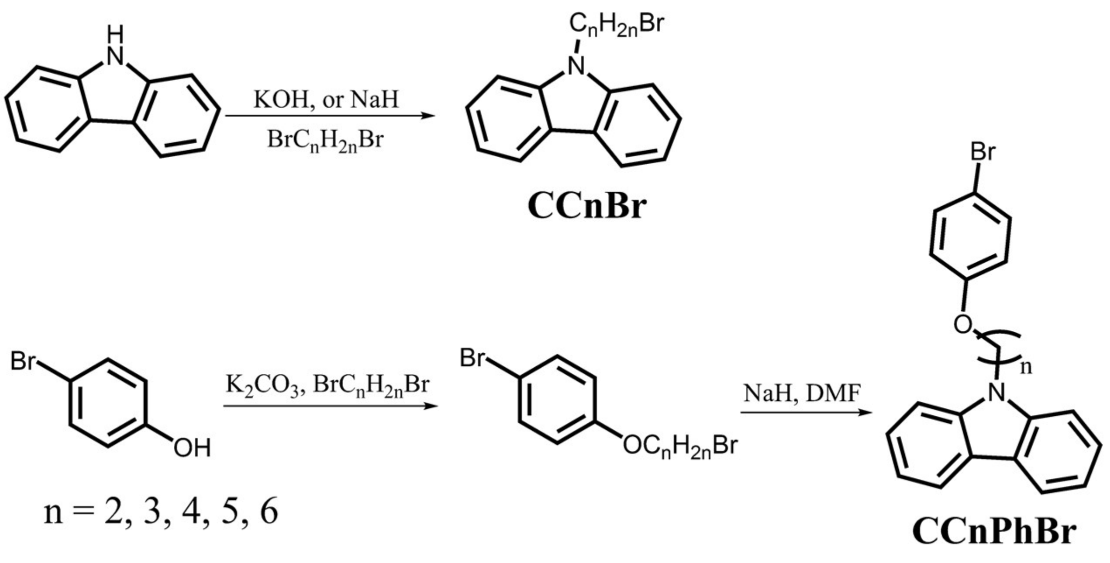

Scheme S1. Synthesis route of **CCnBr** and **CCnPhBr**.

9-(2-bromoethyl)-9H-carbazole (CC2Br)

Carbazole (1.0 g, 6.0 mmol) and 1,2-dibromoethane (10 mL) were dissolved in acetone, and then K_2CO_3 (1.8 g, 13 mmol), KOH (2.2 g, 40 mmol) and Bu_4NBr (40 mg, 0.12 mmol) were added. The mixture was refluxed for 6h, and cooled to room temperature. CH_2CH_2 was added and organic phase was washed by water for three times. Organic layer was dried over anhydrous Na_2SO_4 and removed. Residue was purified by column chromatography (petroleum ether/CH_2Cl_2 = 2/1). White solid as product was obtained in a yield of 23%. mp = 147-148 °C. Elemental analysis: C, 61.33; H, 4.41; N, 5.11; found: C, 61.31; H, 4.45; N, 5.14. ^{1}H NMR (400 MHz, $CDCl_3$) δ 8.13 (d, J = 7.8 Hz, 2H), 7.51 (ddd, J = 8.1, 7.0, 1.1 Hz, 2H), 7.46 (d, J = 8.0 Hz, 2H), 7.29 (ddd, J = 8.1, 7.0, 1.1 Hz, 2H), 4.74 (t, J = 7.6 Hz, 2H), 3.75 – 3.65 (t, J = 7.6 Hz, 2H). ^{13}C NMR (101 MHz, $CDCl_3$) δ 140.07, 126.09, 123.28, 120.68, 119.72, 108.56, 44.80, 28.22. HRMS (m/z): $[M+H]^+$ calcd. for $C_{14}H_{13}BrN$, 274.0226; found, 274.0226.

9-(3-bromopropyl)-9H-carbazole (CC3Br)

Carbazole (1.0 g, 6.0 mmol) and 1,3-dibromopropane (6.0 g, 30 mmol) were dissolved in DMF, and then KOH (1.6 g, 28.5 mmol) was added. The mixture was stirred for 24 h at room temperature, and poured into water. The mixture was extracted with CH_2Cl_2 and the organic layer was washed by water and dried over Na_2SO_4. Colorless oil was obtained from column chromatography (petroleum ether/CH_2Cl_2 = 2/1) after evaporating solvent (38% in yield). Elemental analysis: C, 62.52; H, 4.90; N, 4.86; found: C, 62.55; H, 4.88; N, 4.83. ^{1}H NMR (400 MHz, $CDCl_3$) δ 8.13 (d, *J* = 7.8 Hz, 2H), 7.55 – 7.47 (m, 4H), 7.31 – 7.24 (m, 2H), 4.54 (t, *J* = 6.5 Hz, 2H), 3.42 (t, *J* = 6.2 Hz, 2H), 2.48 (p, *J* = 6.4 Hz, 2H). ^{13}C NMR (101 MHz, $CDCl_3$) δ 140.49, 125.97, 123.12, 120.57, 119.28, 108.74, 41.06, 32.11, 30.99. HRMS (m/z): $[M+H]^+$ calcd. for $C_{15}H_{15}BrN$, 288.0382; found, 288.0375.

9-(4-bromobutyl)-9H-carbazole (CC4Br)

CC4Br was synthesized by the procedure similar to **CC3Br**. mp = 110-111 °C. Elemental Analysis: C, 63.59; H, 5.34; N, 4.63; found: C, 63.62; H, 5.35; N, 4.61. ^{1}H

NMR (400 MHz, $CDCl_3$) δ 8.13 (d, *J* = 7.8 Hz, 2H), 7.51 (ddd, J = 8.1, 7.0, 1.1 Hz, 2H), 7.43 (d, *J* = 8.2 Hz, 2H), 7.29 (ddd, J = 8.1, 7.0, 1.1 Hz, 2H), 4.39 (t, *J* = 6.9 Hz, 2H), 3.41 (t, *J* = 6.5 Hz, 2H), 2.15 – 2.04 (m, 2H), 2.00 – 1.90 (m, 2H). ^{13}C NMR (101 MHz, $CDCl_3$) δ 140.43, 125.86, 123.05, 120.57, 119.10, 108.67, 42.30, 33.27, 30.38, 27.80. HRMS (m/z): $[M+H]^+$ calcd. for $C_{16}H_{17}BrN$, 302.0539; found, 302.0528.

9-(5-bromopentyl)-9H-carbazole (CC5Br)

CC5Br was synthesized by the procedure similar to **CC3Br**. The crude product was purified by column chromatography (petroleum ether/CH_2Cl_2 = 6/1). mp = 61-62 °C. Elemental Analysis: C, 64.57; H, 5.74; N, 4.43; found: C, 64.52; H, 5.75; N, 4.44. ^1H NMR (400 MHz, $CDCl_3$) δ 8.10 (d, *J* = 7.8 Hz, 2H), 7.47 (ddd, J = 8.1, 7.0, 1.1 Hz, 2H), 7.40 (d, *J* = 8.2 Hz, 2H), 7.23 (ddd, J = 8.1, 7.0, 1.1 Hz, 2H), 4.33 (t, *J* = 7.2 Hz, 2H), 3.35 (t, *J* = 6.7 Hz, 2H), 1.97 – 1.82 (m, 4H), 1.59 – 1.46 (m, 2H). ^{13}C NMR (101 MHz, $CDCl_3$) δ 140.47, 125.79, 123.00, 120.53, 118.98, 108.69, 42.94, 33.45, 32.60, 28.32, 26.04. HRMS (m/z): $[M+H]^+$ calcd. for $C_{17}H_{19}BrN$, 316.0695; found, 316.0693.

9-(6-bromohexyl)-9H-carbazole (CC6Br)

CC6Br was synthesized by the procedure similar to **CC3Br**. The crude product was purified by column chromatography (petroleum ether/CH_2Cl_2 = 5/1). mp = 62-63 °C. Elemental Analysis: C, 65.46; H, 6.10; N, 4.24; found: C, 65.42; H, 6.08; N, 4.27. ^1H NMR (400 MHz, $CDCl_3$) δ 8.13 (d, *J* = 7.8 Hz, 2H), 7.49 (ddd, J = 8.1, 7.0, 1.1 Hz, 2H), 7.43 (d, *J* = 8.1 Hz, 2H), 7.26 (ddd, J = 8.1, 7.0, 1.1 Hz, 2H), 4.35 (t, *J* = 7.1 Hz, 2H), 3.39 (t, *J* = 6.7 Hz, 2H), 1.93 (dt, *J* = 14.6, 7.2 Hz, 2H), 1.84 (dt, *J* = 14.6, 7.2 Hz, 2H), 1.54 – 1.37 (m, 4H). ^{13}C NMR (101 MHz, $CDCl_3$) δ 140.53, 125.76, 122.98, 120.52, 118.92, 108.73, 43.00, 33.86, 32.71, 28.97, 28.07, 26.62. HRMS (m/z): $[M+H]^+$ calcd. for $C_{18}H_{21}BrN$, 330.0852; found, 330.0852.

9-(2-(4-bromophenoxy)ethyl)-9H-carbazole (CC2PhBr)

4-bromophenol (1.0 g, 5.8 mmol), 1,2-dibromoethane (3.3 g, 16.8 mmol), K_2CO_3 (1.6

g, 11.6 mmol) and KI (10 mg) was dispersed in acetone and the mixture was refluxed for 24 h. Solid was removed by filtration. The intermediate as colorless oil was obtained by column chromatography (petroleum ether/CH_2Cl_2 = 1/1). Carbazole (1.0 g, 6.0 mmol) was dissolved in DMF, and NaH (0.72 g, 60%) was added slowly. The intermediate (1.5 g in 5 mL DMF) was added dropwise into the above mixture. The mixture was stirred for 5 h at room temperature and poured into water. The mixture was extracted by CH_2Cl_2 and the organic layer was washed by water for three times and dried over anhydrous Na_2SO_4. White solid was obtained by column chromatography (petroleum ether/CH_2Cl_2 = 5/1) in a yield of 19%. mp = 145-147 °C. Elemental Analysis: C, 65.59; H, 4.40; N, 3.82; found: C, 65.64; H, 4.37; N, 3.80. ^{1}H NMR (400 MHz, $CDCl_3$) δ 8.13 (d, J = 7.8 Hz, 2H), 7.54 – 7.47 (m, 4H), 7.33 (d, J = 9.0 Hz, 2H), 7.3 – 7.25 (m, 2H), 6.70 (d, J = 9.0 Hz, 2H), 4.74 (t, J = 5.9 Hz, 2H), 4.34 (t, J = 5.9 Hz, 2H). ^{13}C NMR (101 MHz, $CDCl_3$) δ 157.54, 140.64, 132.40, 125.93, 123.20, 120.56, 119.42, 116.35, 113.44, 108.82, 66.32, 42.56. HRMS (m/z): $[M+H]^+$ calcd. for $C_{20}H_{17}BrNO$, 366.0488; found, 366.0475.

9-(3-(4-bromophenoxy)propyl)-9H-carbazole (CC3PhBr)

By following the synthetic procedure for **CC2PhBr**, **CC3PhBr** as colorless oil was synthesized. Yield: 32%. Elemental Analysis: C, 66.33; H, 4.77; N, 3.68; found: C, 66.31; H, 4.78; N, 3.65. ^{1}H NMR (400 MHz, $CDCl_3$) δ 8.13 (d, J = 7.6 Hz, 2H), 7.39 (d, J = 8.2 Hz, 2H), 7.26 (m, 4H), 6.77 (d, J = 8.1 Hz, 2H), 4.58 (d, J = 12.3 Hz, 2H), 3.88 (s, 2H), 2.37 (d, J = 11.2 Hz, 2H). ^{13}C NMR (101 MHz, $CDCl_3$) δ 157.85, 140.55, 132.43, 125.90, 123.03, 120.50, 119.14, 116.45, 113.20, 108.66, 64.86, 39.41, 28.83. HRMS (m/z): $[M+H]^+$ calcd. for $C_{21}H_{19}BrNO$, 380.0645; found, 380.0635.

9-(4-(4-bromophenoxy)butyl)-9H-carbazole (CC4PhBr)

By following the synthetic procedure for **CC2PhBr**, **CC4PhBr** as colorless oil was synthesized. Yield: 19%. mp = 97-99 °C. Elemental Analysis: C, 67.01; H, 5.11; N, 3.55; found: C, 67.04; H, 5.06; N, 3.58. ^{1}H NMR (400 MHz, $CDCl_3$) δ 8.14 (d, J = 7.8 Hz, 2H), 7.49 (ddd, J = 8.1, 7.0, 1.1 Hz, 2H), 7.44 (d, J = 8.1 Hz, 2H), 7.37 (d, J = 9.0 Hz, 2H), 7.26 (ddd, J = 8.1, 7.0, 1.1 Hz, 2H), 6.75 (d, J = 9.0 Hz, 2H), 4.43 (t, J = 7.0 Hz, 2H), 3.93 (t, J = 6.1 Hz, 2H), 2.18 – 2.05 (m, 2H), 1.87 (m, 2H). ^{13}C NMR (101

MHz, $CDCl_3$) δ 157.98, 140.38, 132.28, 125.70, 122.92, 120.45, 118.91, 116.27, 112.88, 108.63, 67.73, 42.71, 26.96, 25.84. HRMS (m/z): $[M+H]^+$ calcd. for $C_{22}H_{21}BrNO$, 394.0801; found, 394.0807.

9-(5-(4-bromophenoxy)pentyl)-9H-carbazole (CC5PhBr)

By following the synthetic procedure for **CC2PhBr**, **CC5PhBr** as colorless oil was synthesized. Yield: 26%. mp = 92-94 °C. Elemental Analysis: C, 67.65; H, 5.43; N, 3.43; found: C, 67.68; H, 5.41; N, 3.45. ^1H NMR (400 MHz, $CDCl_3$) δ 8.10 (d, J = 7.7 Hz, 2H), 7.46 (ddd, J = 8.1, 7.0, 1.1 Hz, 2H), 7.40 (d, J = 8.1 Hz, 2H), 7.34 (d, J = 9.0 Hz, 2H), 7.23(ddd, J = 8.1, 7.0, 1.1 Hz, 2H), 6.71 (d, J = 9.0 Hz, 2H), 4.34 (t, J = 7.1 Hz, 2H), 3.86 (t, J = 6.4 Hz, 2H), 2.03 – 1.89 (m, 2H), 1.84 – 1.74 (m, 2H), 1.59 – 1.53 (m, 2H). ^{13}C NMR (101 MHz, $CDCl_3$) δ 158.09, 140.41, 132.23, 125.66, 122.87, 120.41, 118.83, 116.30, 112.74, 108.61, 67.85, 42.92, 29.01, 28.79, 23.87. HRMS (m/z): $[M+H]^+$ calcd. for $C_{23}H_{23}BrNO$, 408.0958; found, 408.0940.

9-(6-(4-bromophenoxy)hexyl)-9H-carbazole **(CC6PhBr)**

By following the synthetic procedure for **CC2PhBr**, **CC6PhBr** as colorless oil was synthesized. Yield: 25%. mp = 96-97 °C. Elemental Analysis: C, 68.25; H, 5.73; N, 3.32; found: C, 68.28; H, 5.70; N, 3.34. ^1H NMR (400 MHz, $CDCl_3$) δ 8.13 (d, J = 7.7 Hz, 2H), 7.49 (ddd, J = 8.1, 7.0, 1.1 Hz, 2H), 7.43 (d, J = 8.1 Hz, 2H), 7.37 (d, J = 9.0 Hz, 2H), 7.26(ddd, J = 8.1, 7.0, 1.1 Hz, 2H), 6.74 (d, J = 9.0 Hz, 2H), 4.35 (t, J = 7.1 Hz, 2H), 3.89 (t, J = 6.4 Hz, 2H), 1.99 – 1.91 (m, 2H), 1.79 – 1.69 (m, 2H), 1.49 (m, 4H). ^{13}C NMR (101 MHz, $CDCl_3$) δ 158.14, 140.44, 132.22, 125.63, 122.86, 120.40, 118.79, 116.29, 112.66, 108.64, 67.97, 42.94, 29.02, 28.95, 27.06, 25.92. HRMS (m/z): $[M+H]^+$ calcd. for $C_{24}H_{25}BrNO$, 422.1114; found, 422.1110.

Reference:

1. B. Zhao, X. Zhu, S. Guan, D. Li, *Acta Crystallogr. Sect. E Struct. Rep. Online* **2012**, 68 o2026.

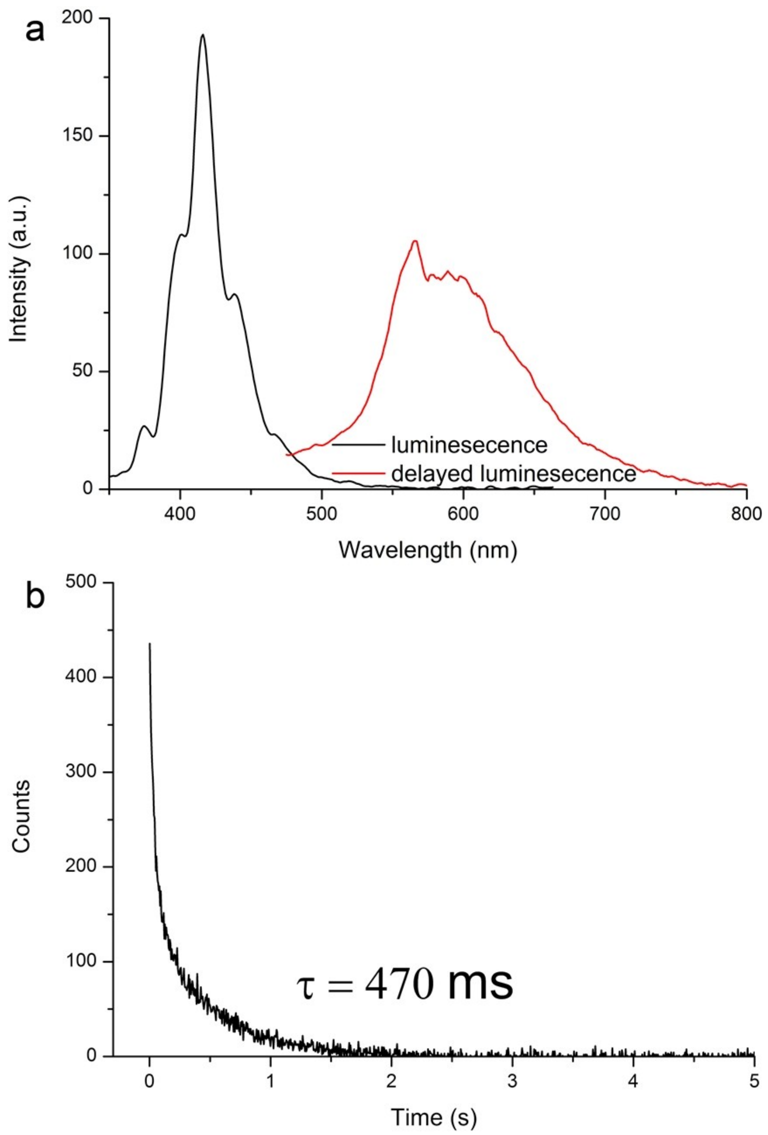

Fig. S1 a) Fluorescence and phosphorescence spectra of carbazole crystal and b) time-resolved emission spectrum at 550 nm. λ_{ex} = 320 nm.

Table S1. Photophysical data of **CCnBr** and **CCnPhBr** in solution and crystal.

	λ_{abs} (solution, nm)	λ_{abs} (solid, nm)	λ_{em} (solution, nm)	Φ (solution)	λ_{em} (solid, nm)	Φ (%) (solid)	τ (solid, lifetime)
**CC2Br**	327; 341	335; 350	341; 357	0.18	370; 384	1.1	0.96 ns; (370 nm)
					407; 430; 455		3.4 ns; (407 nm)
					544; 591; 644	1.6	160 ms
**CC4Br**	331; 345.5	335; 350	345; 362	0.45	374.5; 388	14.3	2.26ns; (374 nm)
					414; 437; 468		2.78 ns; (414 nm)
					555.5; 604.5; 661	11.1	85ms
**CC5Br**	332; 346	337; 351	346; 362	0.57	369; 382;	38.4	6.39 ns (370 nm)
					544; 585; 635	2.1	20ms
**CC6Br**	332; 346.5	335; 350	346; 362	0.47	375; 388; 413	46.5	7.08ns (375 nm)
**CC2PhBr**	328; 342	334; 348	342; 358	0.43	371; 383; 390	25.2	1.58 ns (370 nm)
					410; 436; 462		4.42ns (410 nm)
					554; 602; 660	8.9	64ms
**CC4PhBr**	331; 345.5	334; 349.5	346; 364	0.37	373; 388;	26.1	4.79ns (373 nm)
					415; 438; 467		9.68 ns (415 nm)
					555; 605; 660	9.5	340 ms
**CC5PhBr**	331.5; 346	334; 350	346; 362	0.40	373; 359	8.38	1.6 ns (373 nm)
					414; 440;		4.02 (414 nm)
					555; 604;	2.75	197 ms
**CC6PhBr**	332; 346.5	336.5; 352	347; 362	0.45	376; 394;	33.1	3.90 ns; (370 nm)
					420; 440; 470		11.78 ns (415 nm)
					554; 602; 659	39.47	200 ms

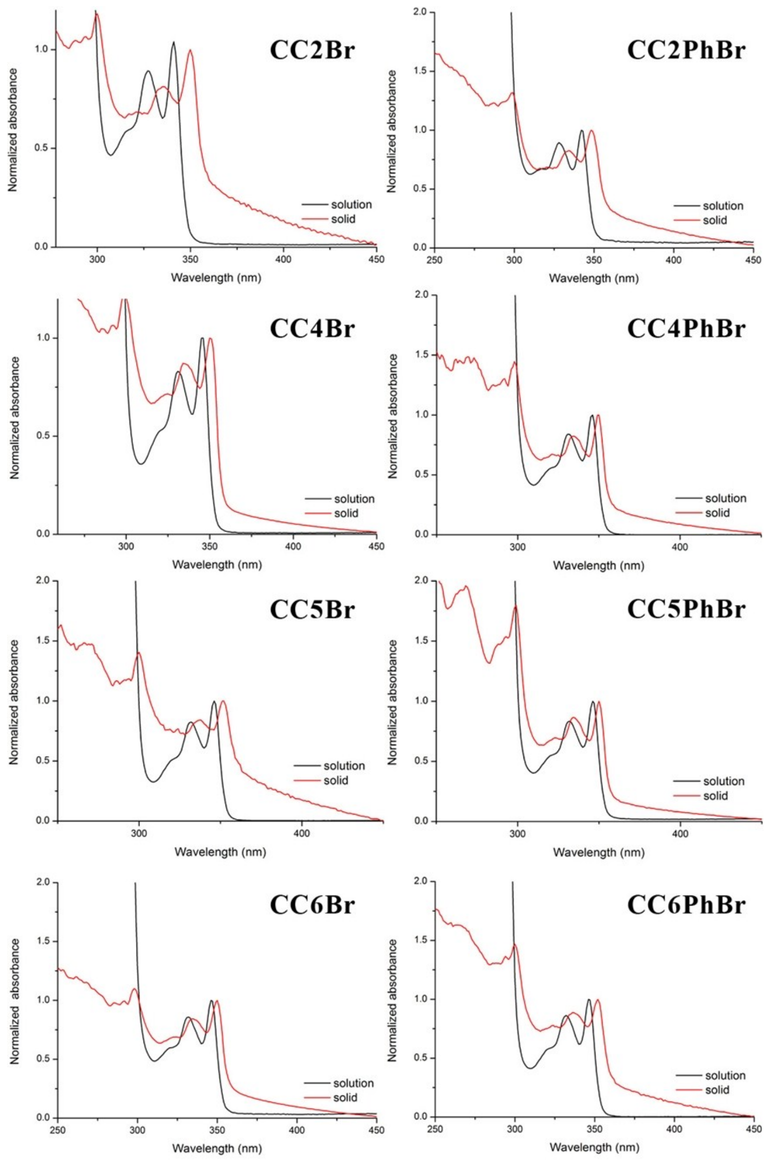

Fig. S2 Normalized UV-vis absorption spectra of **CCnBr** and **CCnPhBr** in toluene solutions (1.0×10^{-5} M) and in crystal state.

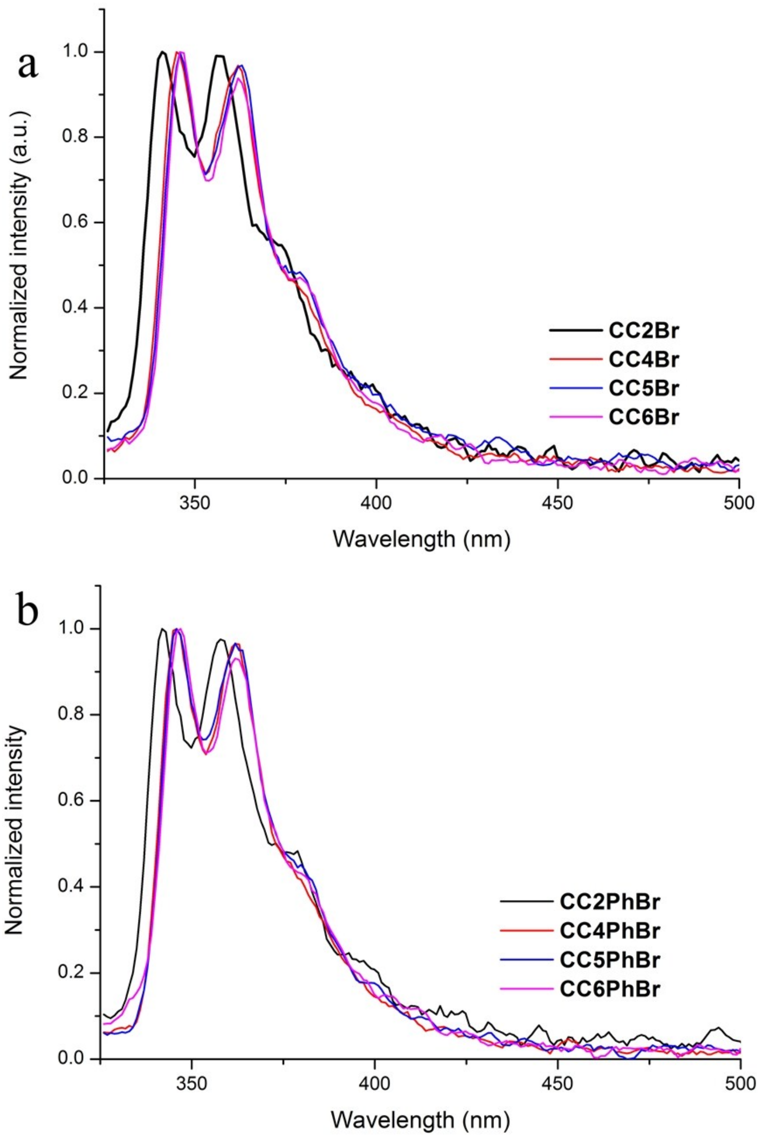

Fig. S3 Luminescence spectra of (a) **CCnBr** and (b) **CCnPhBr** in cyclohexane (10^{-5}M). λ_{ex} = 280 nm.

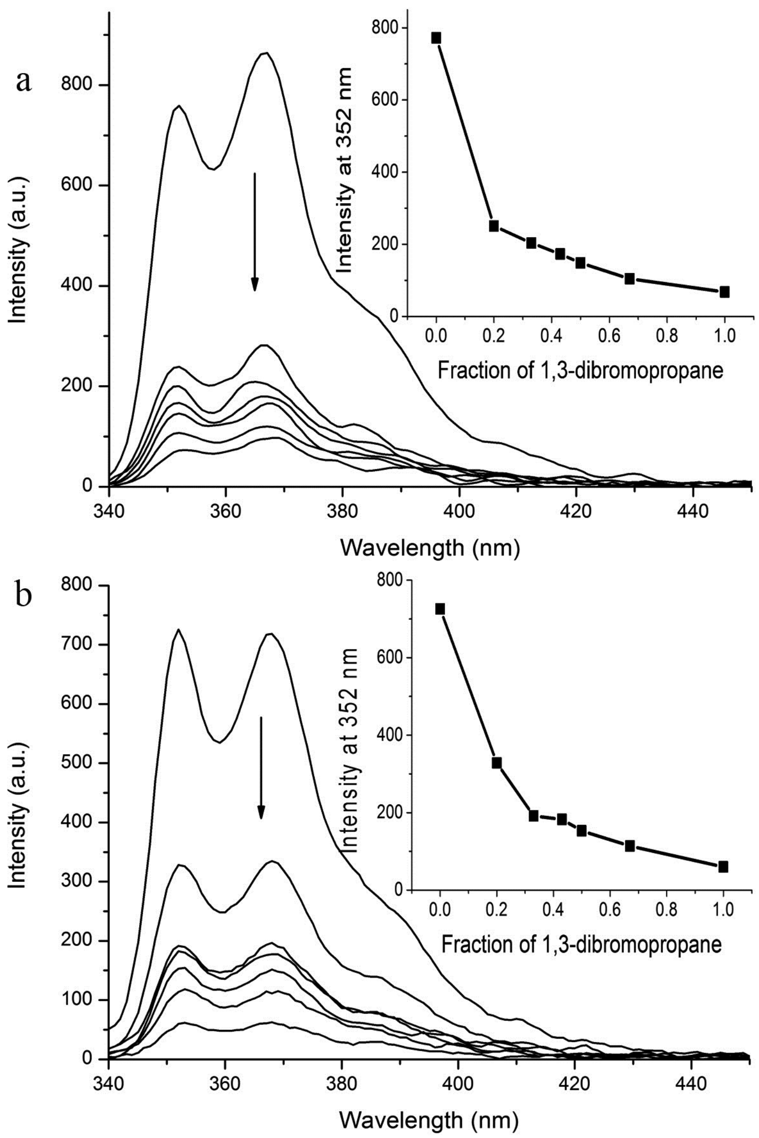

Fig. S4 Fluorescence spectra of (a) **CC4Br** and (b) **CC4PhBr** in the mixtures of CH_2Cl_2 and 1,3-dibromopropane with different volume ratios. λ_{ex} = 280 nm.

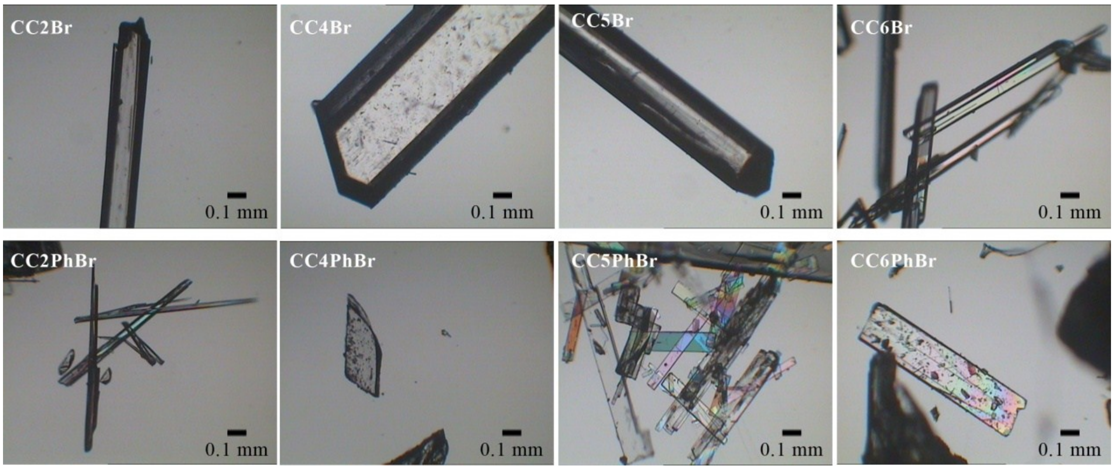

Fig. S5 Optical microscopy images of crystals.

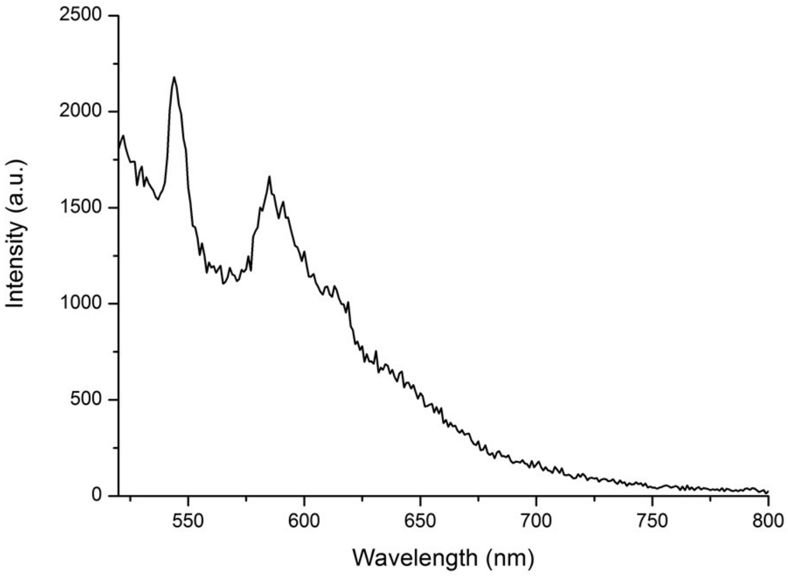

Fig. S6 PL spectrum of **CC5Br** crystal with a larger slit. λ_{ex} = 280 nm.

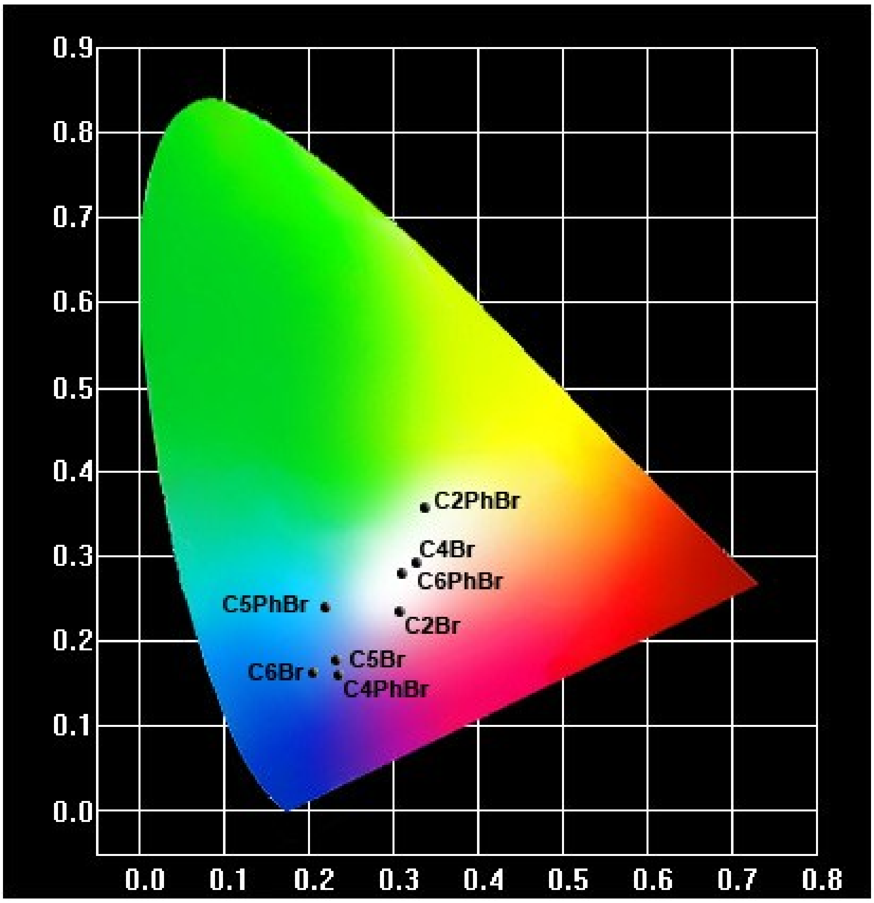

Fig. S7 CIE chromaticity coordinates of eight compounds.

Table S2. CIE coordinates of eight compounds.

Compounds	CIE	Compounds	CIE
**CC2Br**	0.31, 0.24	**CC2PhBr**	0.30, 0.27
**CC4Br**	0.33, 0.30	**CC4PhBr**	0.24, 0.16
**CC5Br**	0.24, 0.18	**CC5PhBr**	0.23, 0.24
**CC6Br**	0.21, 0.16	**CC6PhBr**	0.31, 0.28

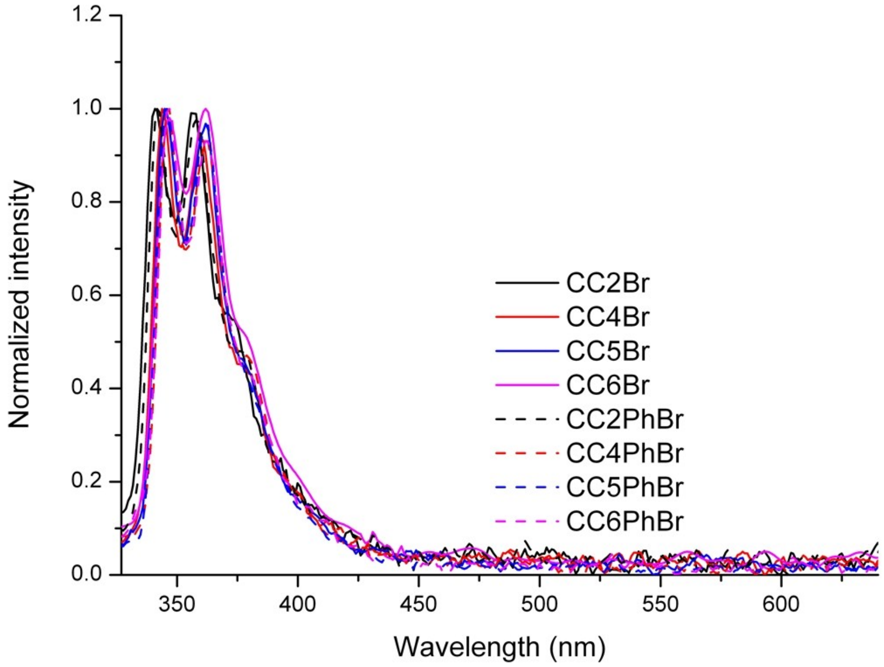

Fig. S8 Photoluminescence spectra of PMMA film doped with **CCnBr** and **CCnPhBr** (5.0% in weight). λ_{ex} =280 nm.

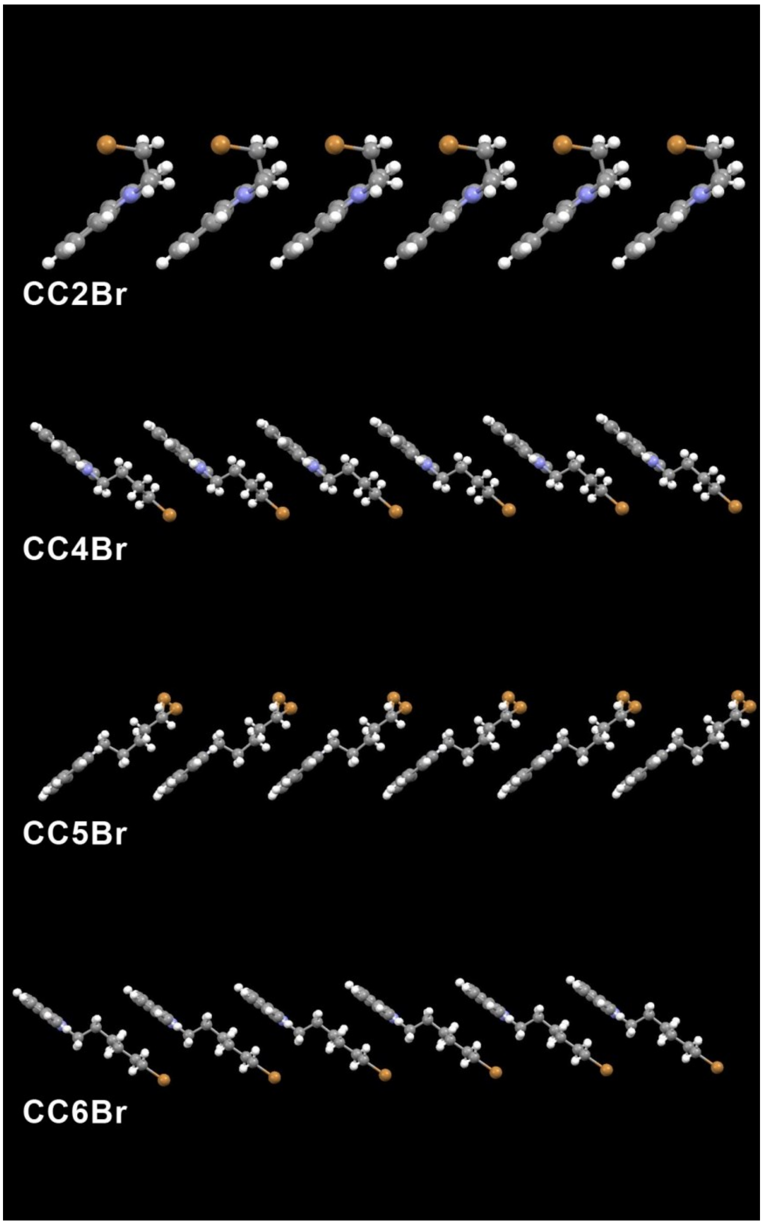

Fig. S9 1D aggregates of **CCnBr** in crystals.

Table S3. Short interactions in **CCnBr** crystals.

Compounds	Short interactions	Distance (Å)
**CC2Br**	2, 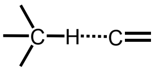	2.832
**CC4Br**	2, 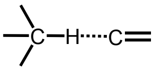	2.820
	2, =C—H·····C=	2.854
**CC5Br**	2, =C—H·····C=	2.840
	2, 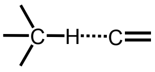	2.805
	2, C-H···Br	2.995
	Br···**Br**	3.693
**CC6Br**	2, 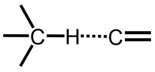	2.883
	2, 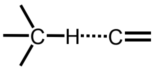	2.854

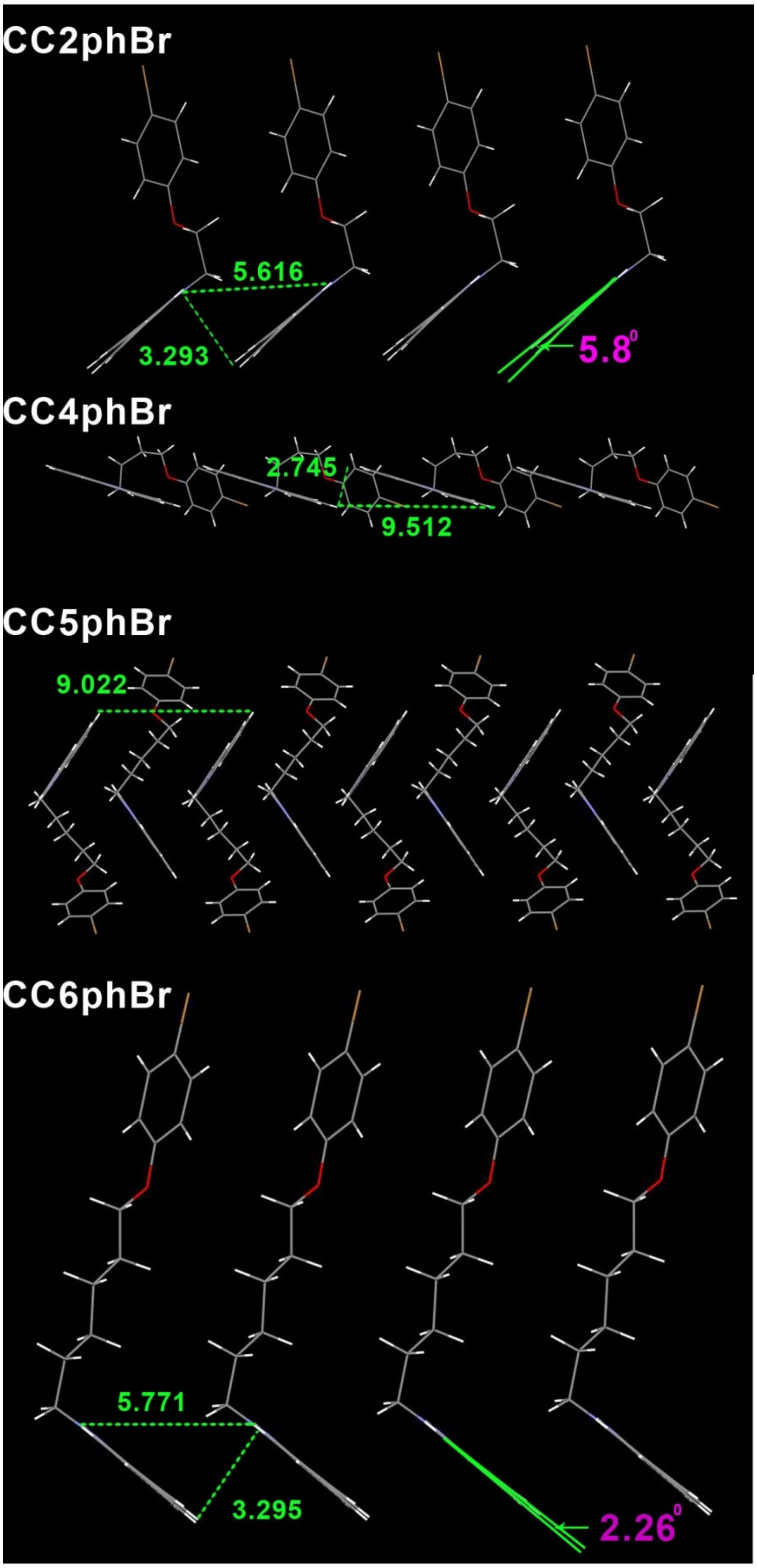

Fig. S10 1D aggregates with the distance and interplanar spacing between adjacent carbazoles in **CCnPhB**r crystals.

Table S4. Short interactions in **CCnPhBr** crystals.

Compounds	Short interactions	Distance (Å)
**CC2PhBr**	2, =C–H····C=	2.756
	2, =C–H····C=	2.828
	2, >C–H····C=	2.720
	2, >C······C=	3.332
	2, =C–H····C=	2.809
**CC4PhBr**	2, >C–H····C=	2.895
	2, =C–H····C=	2.842
	2, =C-H···Br	3.028
**CC5PhBr**	2, >C–H····C=	2.884
	2, >C–H····C=	2.813
	2, >C–H····C=	2.829
	2, =C–H····C=	2.893
	2, >C–H····C=	2.844
	2, =C–H····C=	2.867
**CC6PhBr**	2, >C······C=	3.389
	2, >C–H····C=	2.797
	2, >C–H····C=	2.826
	2, =C–H····C=	2.773
	2, >C–H····H–C=	2.373
	2, >C–H····H–C=	2.310
	2, >C······C=	2.892

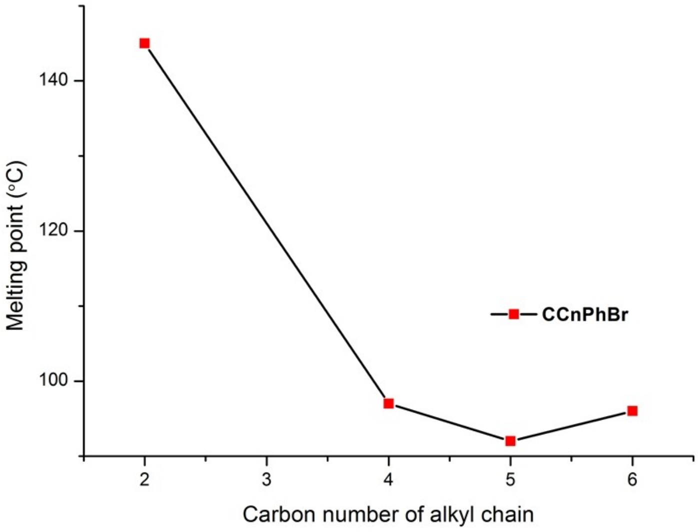

Fig. S11 Melting points of **CCnPhBr**.
